# Vibrational Spectroscopy and Computational Studies of Cubane-1,4-Dicarboxylic Acid

**DOI:** 10.3390/molecules31040592

**Published:** 2026-02-09

**Authors:** Stewart F. Parker, James P. Tellam, Sarah E. Youngs

**Affiliations:** ISIS Neutron and Muon Facility, STFC Rutherford Appleton Laboratory, Chilton OX11 0QX, Oxon, UK; james.tellam@stfc.ac.uk (J.P.T.); sarah.youngs@stfc.ac.uk (S.E.Y.)

**Keywords:** inelastic neutron scattering, infrared spectroscopy, Raman spectroscopy, density functional theory, cubane-1,4-dicarboxylic acid

## Abstract

Cubane-1,4-dicarboxylic acid is a key intermediate in the synthesis of the Platonic solid, cubane. While cubane has been extensively studied, its precursor has not. Here, we provide a comprehensive characterization of the vibrational spectra (infrared, Raman, inelastic neutron scattering (INS)) of cubane-1,4-dicarboxylic acid and its isotopomer with the acidic hydrogens exchanged for deuterium. In combination with density functional theory studies of the complete unit cell, we show that the dynamics of the carboxylic acid and the cubane core are largely independent; the effect is mostly the result of the increased mass of the substituent at the 1,4 positions: 45 versus 1. The known crystal structure is unusual in that the carboxylic acid is present as two conformers: syn and anti. The calculations show that the in-plane and out-of-plane C–O–H bending modes have different transition energies in the two conformers. For all the other modes, both conformers contribute approximately equally.

## 1. Introduction

**Figure 1:**
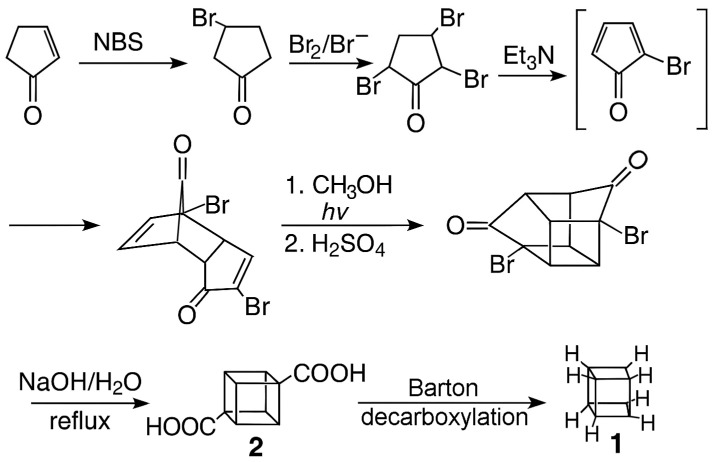
The currently used synthesis of cubane (NBS = N-bromosuccinimide). Adapted from [2] with the permission of John Wiley and Sons.

Cubane (**1**, C_8_H_8_, pentacyclo[4.2.0.0^2,5^.0^3,8^.0^4,7^]octane; see Figure 1 for the structure) is the simplest of the Platonic solids to have been synthesized [1,2] in an unsubstituted form. (Tetrahedrane, C_4_H_4_, the simplest Platonic solid, is only known as a tetrasubstituted derivative [3,4]). Starting from 2-cyclopentenone, the original synthesis in 1964 involved 12 steps, with the final step being the decarboxylation of the tertiary butyl perester of cubanecarboxylic acid [1,5]. This has evolved into the seven-step synthesis shown in Figure 1 [2,6], with the final step being the photodecarboxylation of cubane-1,4-dicarboxylic acid (the Barton reaction [7]). Crucially, the final intermediate, cubane-1,4-dicarboxylic acid, **2**, is now commercially available from several sources [8,9,10,11].

Both cubane [12,13] and cubane-1,4-dicarboxylic acid [14] have been characterized in the solid state by single crystal X-ray diffraction. Although cubane occupies a site with *C*_3i_ symmetry, the molecule is cubic, with all C–C distances lying between 1.571 and 1.572 Å and all internal ∠C–C–C being within 0.01° of 90° and each of the six cubane four-carbon-atom faces is a perfect square, flat within 0.0001 Å [13]. In cubane-1,4-dicarboxylic acid, the molecule occupies a general site and thus has *C*_1_ symmetry. The system is also unusual in that there are two conformers present. These differ in terms of how the acidic hydrogen of the carboxylic acid is oriented: they are syn-periplanar where the O–H bond points away from the cubane core, and anti-periplanar where the O–H bond points towards the cubane core. The two conformers are similar, but in both, the cubic symmetry of cubane is relaxed: C–C bond lengths of 1.558 to 1.585 Å (syn) and 1.558 to 1.585 Å (anti) and internal ∠C–C–C of 88.974 to 90.674° (syn) and 88.958 to 90.696° (anti), while the square faces differ from planarity by 0.138 to 1.044° (syn) and 0.159 to 1.921° (anti).

The vibrational spectroscopy of cubane has been extensively investigated both experimentally [15,16,17,18] and computationally [17,18,19]; however, we are unaware of any studies of the vibrational spectroscopy of cubane-1,4-dicarboxylic acid. The aim of this paper is to explore how the effects of the reduction in symmetry from cubane to cubane-1,4-dicarboxylic acid are manifested in the vibrational spectra. The analysis is supported by fully periodic density functional theory calculations.

## 2. Results and Discussion

### 2.1. Vibrational Analysis

The highest possible symmetry of either conformer of cubane-1,4-dicarboxylic acid is *C*_2h_. The crystal has *P*2_1_/c symmetry with lattice parameters *a* = 7.2512 (6) Å, *b* = 12.9050 (12) Å, *c* = 8.3031 (5) Å and β = 90.993 (6)°. The centre of mass of each of the two conformers occupy sites of *C*_i_ symmetry, Wyckoff site *a* for the syn conformer, and site *c* for the anti conformer. Each Wyckoff site has a multiplicity of two; hence, *Z* = 4. The correlation table [20] for the system is given in Table 1.

It can be seen that because the site group preserves the centre of symmetry, modes that are gerade (g) in the free molecule retain the same designation in the crystal. The same applies to the ungerade (u) modes in the free molecule and in the crystal. Also, each mode in the free molecule gives rise to two pairs of modes in the crystal. The temptation is to assume that one pair relates to each conformer, i.e., that each conformer behaves independently of the other. We will show that this assumption is only partly valid.

### 2.2. Vibrational Spectra

**Figure 2:**
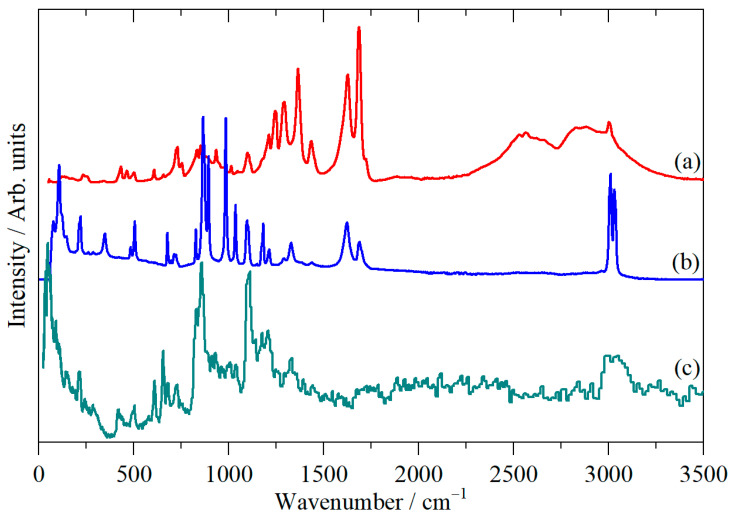
Vibrational spectra of cubane-1,4-dicarboxylic acid: (**a**) infrared, (**b**) FT-Raman and (**c**) INS. (**a**,**b**) were measured at room temperature and (**c**) at 10 K.

**Figure 3:**
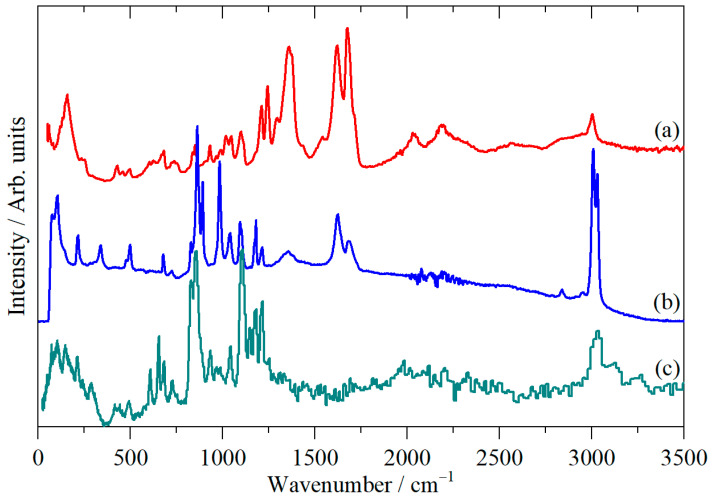
Vibrational spectra of cubane-1,4-dicarboxylic acid-D2: (**a**) infrared, (**b**) FT-Raman and (**c**) INS. (**a**,**b**) were measured at room temperature and (**c**) at 10 K.

Figure 2 shows the infrared, Raman, and inelastic neutron scattering (INS) spectra of cubane-1,4-dicarboxylic acid and Figure 3 the spectra after the carboxylic acid protons were exchanged for deuterium (cubane-1,4-dicarboxylic acid-D2).

INS spectroscopy [21] is a form of vibrational spectroscopy that is analogous to Raman spectroscopy in that it is an inelastic scattering process; the difference is that the scattering particle is a neutron in INS and a photon in Raman spectroscopy. A key difference between INS and optical spectroscopies is that INS has no selection rules, so all modes are allowed and, in principle, observable. However, the intensity of an INS mode depends on the amplitude of motion of the atoms in the mode and their scattering cross-section. ^1^H is the lightest isotope (hence it has the largest amplitude of motion) and has a scattering cross-section ~20-fold larger than most other elements. Thus, while there are no selection rules, there is a very strong propensity for modes involving hydrogen motion to dominate the INS spectrum. This is apparent in Figure 2 and Figure 3, where the carbonyl stretching modes between 1500 and 1750 cm^−1^ are not visible in the INS but give strong features in the infrared and Raman spectra. Conversely, some of the modes below 800 cm^−1^ give strong features in the INS that are barely visible in the infrared and Raman spectra. For the type of instrument used here (TOSCA, an indirect geometry spectrometer [21]), the spectral range is −25–4000 cm^−1^, but the resolution worsens with increasing wavenumber, so it is optimal in the 16–2000 cm^−1^ regions.

The scattering cross-section is both element- and isotope-dependent and that of deuterium (^2^H) is only ~5% of ^1^H. This means that in addition to the downshift in wavenumber that occurs because of the increased mass, modes involving D do not appear strongly in the INS spectrum.

**Figure 4:**
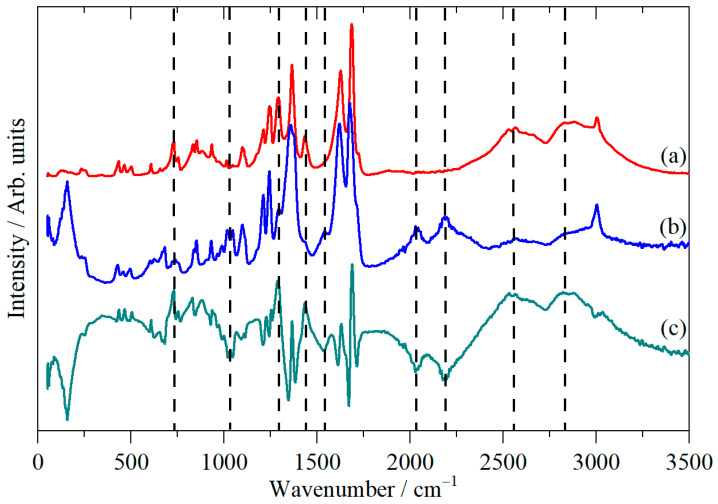
Infrared spectra of (**a**) cubane-1,4-dicarboxylic acid, (**b**) cubane-1,4-dicarboxylic acid-D2, and (**c**) the scaled difference spectrum (**a**,**b**). Modes that show deuterium sensitivity are indicated by vertical dashed lines.

The carboxylic acid O–H and C=O stretch modes are most clearly seen in the infrared spectrum (Figure 2a) at 2500–3000 cm^−1^ and 1500–1750 cm^−1^, respectively, although the latter are also obvious in the Raman spectrum. For the deuterated material (Figure 3a), we see the expected shift in the O–H stretch modes to 1900–2300 cm^−1^. There is a residual peak around 3000 cm^−1^, but it is clear that the material is highly deuterated. The effect of deuteration is most clearly seen in the infrared spectra. Figure 4a,b show these spectra, and a difference spectrum can be seen in Figure 4c, generated by scaled subtraction of the D2 spectrum from the all-H system.

### 2.3. Assignment of the Spectra

Apart from the C–H stretch modes around 3000 cm^−1^, all of cubane’s internal vibrations occur in the range of 600–1250 cm^−1^ [15], so all of the modes between ~200 and 600 cm^−1^ and 1250 and 1750 cm^−1^ must originate from the carboxylic acid functionalities. As there are four molecules in the primitive cell, there will be three acoustic translation modes, nine optic translation modes and twelve librational modes. These will occur below 200 cm^−1^.

**Figure 5:**
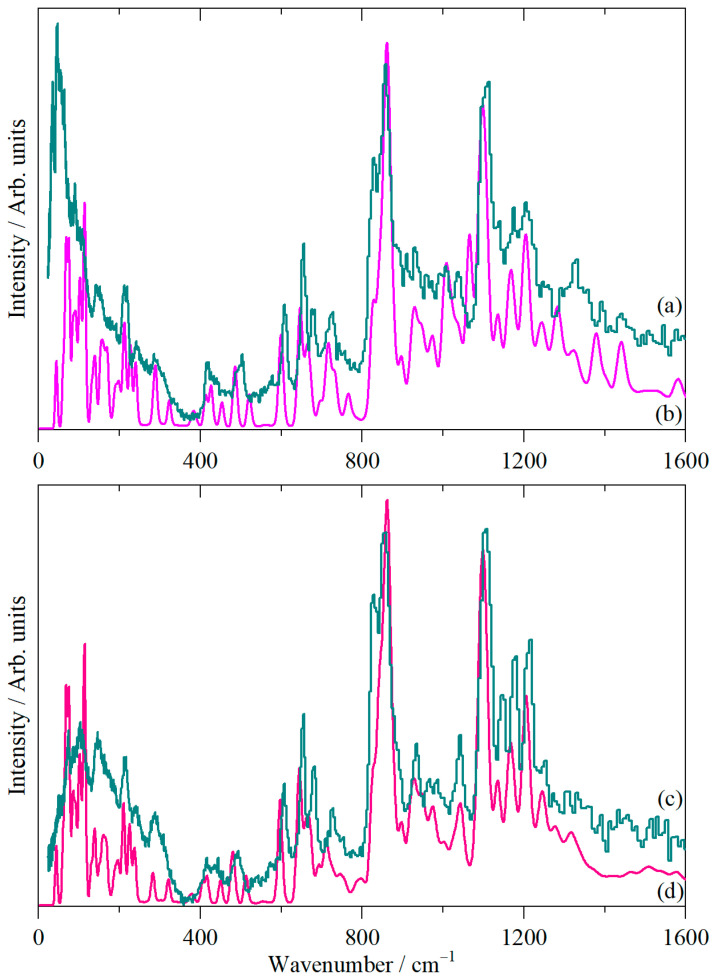
Comparison of observed (**a**,**c**) INS spectra and those generated from a periodic-DFT calculation (**b**,**d**). Top panel: cubane-1,4-dicarboxylic acid. Bottom panel: cubane-1,4-dicarboxylic acid-D2.

Figure 5 compares the experimental INS spectra of cubane-1,4-dicarboxylic acid and cubane-1,4-dicarboxylic acid-D2 with those generated from periodic density functional theory (periodic-DFT) calculations. It can be seen that there is remarkable agreement between the experimental and calculated spectra. The only region where there is significant disagreement is the region below 200 cm^−1^. Here, the modes are largely translations (acoustic and optic) and librations and these are likely to show significant vibrational dispersion (variation in transition energy with wavevector), as the hydrogen bonding provides a mechanism for adjacent unit cells to interact. As INS can observe modes at all wavevectors, these will appear as a broad, structured feature in the INS spectrum, exactly as seen. The only other modes that may show any significant dispersion are the O–H stretch and deformation modes. For instrumental reasons explained elsewhere [21], the O–H stretch region does not provide any useful information in the TOSCA INS spectrum. As all the modes in the 600–1400 cm^−1^ are relatively sharp, this suggests that the dispersion is less than the observed band widths (10–20 cm^−1^). In principle, it is possible to calculate the spectra as a function of the wavevector; however, the size of the current system prohibits this.

**Figure 6:**
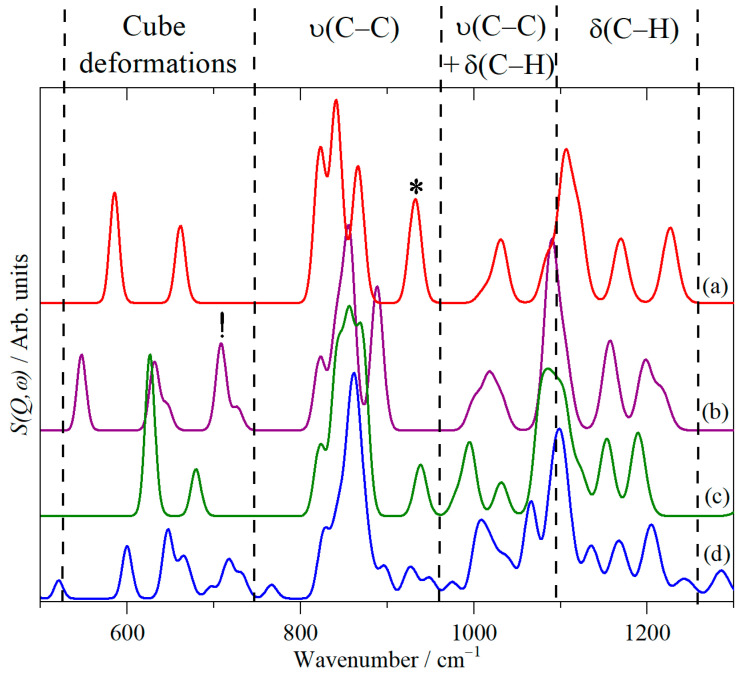
The effect of increasing mass at the 1,4 positions of cubane on the calculated spectra: (**a**) cubane, (**b**) cubane-1,4-D2, (**c**) cubane-1,4-M45 (see text), and (**d**) cubane-1,4-dicarboxylic acid (ν = stretch; δ = bend, the vertical dashed lines define the regions that the indicated modes occur in, * see text, ! indicates a C–D bending mode).

Cubane’s vibrations can be categorized as shown at the top of Figure 6 [15]. The figure shows the effect of increasing mass at the 1,4 positions of cubane: from 1 in (a), to 2 in (b), to 45 in (c) (this corresponds to the mass of the COOH group, “cubane-1,4-M45”), and to 45 in (d), which is cubane-1,4-dicarboxylic acid. It can be seen that the C–H bending modes (δ(C–H)) are largely unchanged, as are the C–C stretch modes (ν(C–C)), except for the mode at 932 cm^−1^ (indicated by *). This involves two opposite faces of the cube moving backwards and forwards; thus, all of the carbon atoms are involved, and therefore, as the effective mass of the carbon atom (i.e., atom + substituent) increases, the transition energy will drop, exactly as seen.

In the cube deformation region, deuterium substitution has only a minor effect (the “new” peak at 708 cm^−1^ marked by “!” is a C–D bending mode). Increasing the mass to 45 unexpectedly results in an upshift in transition energy. The mode animations show that this is because the heavier masses at the 1,4 positions decouple these carbon atoms from the mode. This means that there is less movement of mass in the mode, so the transition energy increases.

The calculated spectrum of cubane-1,4-dicarboxylic acid (Figure 6d) largely mimics that of the mass 45 pseudo-cubane in this region (Figure 6c), demonstrating that the vibrations of the carboxylic acid groups are largely decoupled from those of the cubane core.

The infrared spectra are dominated by the O–H stretch. This has maxima of 2550 and 2860 cm^−1^. Two possible explanations are that they represent differences in hydrogen bond strength between the syn and anti conformers, or that they are the result of Fermi resonance (an anharmonic interaction) between the O–H stretch fundamental and an overtone or combination of the O-H deformation modes that results in intensity transfer from the fundamental to the overtone/combination mode.

There is a well-established correlation between the infrared stretching wavenumber and the O⋯O distance [22]. This predicts distances of 2.56–2.60 and 2.63–2.67 Å for the 2550 and 2860 cm^−1^ modes, respectively. For comparison, the crystallographic distances are 2.627 (anti) and 2.637 (syn) Å, which would predict the modes to be in the range of 2600–3000 cm^−1^. Thus, the crystallography prediction agrees reasonably well with the experimental results, but the spectroscopic prediction is modest at best. This suggests that the two strengths of the hydrogen bonding model are incorrect.

For the Fermi resonance model to be tenable, there must be an O–H deformation mode around 1275 cm^−1^ for an overtone or two modes that bracket this value for a combination. Inspection of Figure 4 shows a deuterium-sensitive mode at 1295 cm^−1^, which the mode animations show to be a mixture of the in-plane O–H deformation and the cubane–COOH stretch. If this mode is considered to be the relevant one, then 2 × 1295 cm^−1^ = 2590 cm^−1^, c.f. 2550 cm^−1^ observed; thus, it would require an anharmonicity constant of 20 cm^−1^ (i.e., ~1.5%, which is reasonable for a hydrogenous mode), assuming that the Fermi resonance does not shift the transition energies (which it may do). The O–H features shift to 2033 and 2188 cm^−1^ on deuterium substitution, and the 1295 cm^−1^ O–H bend shifts to 1032 cm^−1^. For both the 1032 and 2033 cm^−1^ modes, the isotope ratio (H/D) is 1.25, consistent with our argument in favour of Fermi resonance.

The periodic-DFT calculations predict the O–H stretch modes at 2648–2659 and 2754–2805 cm^−1^. The mode animations show that these correspond to the two O–H groups on each molecule moving out-of-phase and in-phase, respectively. Notably, the difference between the two groups is only ~100 cm^−1^, much less than the ~300 cm^−1^ observed. The calculations need to be treated with caution as they are based on the harmonic approximation and the O–H stretch modes will be distinctly anharmonic; however, the difference between the in-phase and out-of-phase modes should be reliable. Additionally, as Fermi resonance is an anharmonic effect, it will not appear in the calculated spectra. Overall, the assignment of the O–H stretch is most consistent with the unresolved fundamental modes being at 2860 cm^−1^, and the 2550 cm^−1^ feature being a Fermi resonance enhanced overtone of the in-plane O–H bending mode. To further support this assignment, fully anharmonic calculations would be required. Unfortunately, this is beyond our current capabilities.

Regarding the C=O stretch modes at 1626 and 1691 cm^−1^, the mode animations show that these are the two carbonyl groups on each molecule moving in-phase and out-of-phase, respectively. In the infrared spectrum, the asymmetric out-of-phase mode is the stronger one, while in the Raman spectrum, it is the symmetric in-phase mode that is stronger.

**Figure 7:**
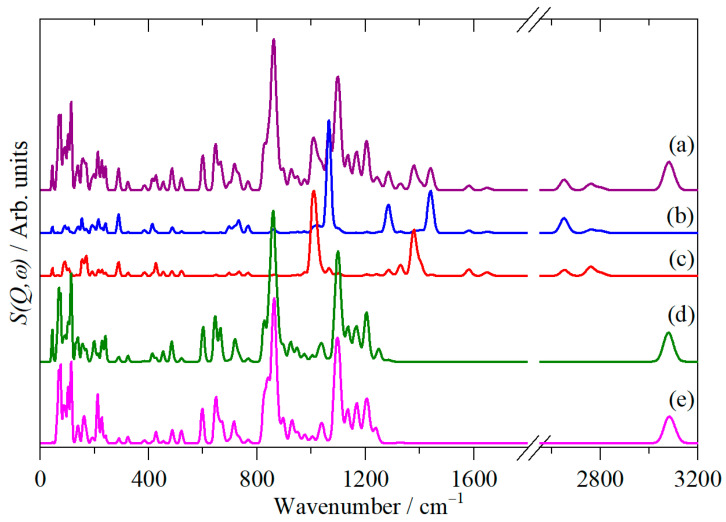
Calculated INS spectra of cubane-1,4-dicarboxylic acid: (**a**) total spectrum comprising contributions from all of the hydrogen atoms; (**b**) contribution of only the carboxylic acid hydrogens of the syn conformer; (**c**) contribution of only the carboxylic acid hydrogens of the anti conformer positions; (**d**) contribution of only the cubane hydrogens of the syn conformer; and (**e**) contribution of only the cubane hydrogens of the anti conformer. Note that (**b**–**e**) are ×2 ordinate expanded relative to (**a**), and that only the fundamentals, i.e., 0 ⟶ 1 vibrational transitions, are shown.

A unique feature of INS spectroscopy is that it is possible to isolate the contribution of an atom or group of atoms to the calculated spectrum. This is because the INS intensity depends, in part, on the amplitude of motion of the atom in the mode [21]. Figure 7 shows the total spectrum and the contributions of only the carboxylic acid hydrogens and the hydrogens of the cubane core for each conformer.

For the carboxylic acid hydrogens (Figure 7b,c) below 800 cm^−1^, the spectra are essentially identical, showing that both conformers contribute more or less equally to all the modes. At higher energy, this is not the case. The out-of-plane bending mode of the carboxylic acid hydrogen is seen at 1006 and 1065 cm^−1^ for the anti and syn conformers, respectively. Similarly, the in-plane bending mode is seen at 1383 cm^−1^ for the anti conformer and 1285 and 1443 cm^−1^ for the syn conformer. There are two modes for the syn system because the C–O–H bend is strongly coupled to the cubane–COOH stretch, and both bands have large contributions from each motion.

In contrast, the spectra of the cubane hydrogens almost overlay each other, showing that both conformers contribute to the modes.

## 3. Materials and Methods

Cubane-1,4-dicarboxylic acid (≥97%) was purchased from Merck (Gillingham, UK) and used as received. Cubane-1,4-dicarboxylic acid-D2 was prepared from the protonated material by stirring in D_2_O (Merck, Gillingham, UK) at 40 °C overnight. The D_2_O was removed by freeze drying. A second cycle using D_2_O was carried out as above. Finally, the crude product was dissolved in CH_3_OD at 50 °C overnight before concentrating in vacuo to afford the product as a white solid. ^1^H and ^13^C NMR spectra showed 96% deuteration. ^1^H and ^13^C NMR spectra of both the as-received and deuterated compounds were measured at room temperature in DMSO-D6 solution using a Bruker (Billerica, MA, USA) Avance NEO 400 MHz NMR spectrometer. The NMR spectra are included in the deposited data.

Room temperature infrared spectra (64 scans at 4 cm^−1^ resolution with eight times zero filling (to improve the peak shape)) were recorded with a Bruker (Billerica, MA, USA) Vertex 70 FTIR spectrometer, over the 50–4000 cm^−1^ range using the Bruker Diamond ATR accessory. Raman spectra were recorded at room temperature with the sample in a quartz cell using a Bruker (Billerica, MA, USA) FT-Raman spectrometer (64 scans at 1 cm^−1^ resolution with 500 mW laser power at 1064 nm and eight times zero filling). For the INS measurements, ~2 g of cubane-1,4-dicarboxylic acid or cubane-1,4-dicarboxylic acid-D2 was loaded into an indium wire-sealed Al can. The sample was quenched in liquid nitrogen immediately before insertion into the indirect geometry, high-resolution, broadband spectrometer TOSCA [23,24] at ISIS [25] and measured for ~3 h. All of the vibrational spectra were recorded from the same batch of material (H or D).

DFT calculations were carried out using the plane-wave, pseudopotential code CASTEP v23.1 [26]. Exchange and correlation were approximated using the Perdew–Burke–Ernzerhof functional [27], with the Tkatchenko–Scheffler dispersion correction scheme [28] within the generalized gradient approximation. On-the-fly-generated norm-conserving pseudopotentials were used. The plane-wave cut-off was 1020 eV, and the Brillouin-zone sampling of electronic states used a 6 × 4 × 6 Monkhorst–Pack grid (36 *k*-points). The equilibrium structure, an essential prerequisite for lattice dynamics calculations, was obtained by Broyden–Fletcher–Goldfarb–Shanno geometry optimization, after which the residual forces were converged to zero within ±0.003 eV Å^−1^. Phonon frequencies were obtained by the diagonalization of dynamical matrices computed using density-functional perturbation theory [29]. An analysis of the resulting eigenvectors was used to map the computed modes to the corresponding irreducible representations of the point group and assign IUPAC symmetry labels. DFPT was also used to compute the dielectric response and the Born effective charges, and from these, the mode oscillator strength tensor and infrared absorptivity were calculated. The spectra of the isotopic species were calculated using the CASTEP utility Phonons [30]. The INS spectra were generated from the CASTEP output using AbINS [31].

## 4. Conclusions

In the present work, we have provided a comprehensive characterization of the vibrational spectra (infrared, Raman, INS) of the cubane precursor cubane-1,4-dicarboxylic acid and its isotopomer with the acidic hydrogens exchanged for deuterium. In combination with density functional theory studies of the complete unit cell, we have demonstrated that the dynamics of the carboxylic acid and the cubane core are largely independent of each other. The changes to the cubane modes are mostly the result of the increased mass of the substituent at the 1,4 positions; in essence, the effect of the carboxylic acid moiety is to behave as an isotope of hydrogen with a mass of 45.

The known crystal structure is unusual in that the carboxylic acid is present as two conformers: syn and anti. The calculations show that only the in-plane and out-of-plane C–O–H bending modes have different transition energies in the two conformers; these are the only modes where each pair of conformers behave independently. For all the other modes, both conformers contribute approximately equally, i.e., they are collective modes involving all four molecules in the unit cell.

This work vividly illustrates the powerful synergy of computational studies and INS. The excellent agreement between the observed and calculated INS spectra demonstrates that the model accurately reproduces the dynamics of the system, validating the calculations. These can then be interrogated to isolate the contributions of an individual atom (or atoms) to the spectrum. These insights are only possible because of the unique characteristics of INS—in particular, that the intensity depends only on the motion of the atom(s); the electronic response plays no part.

## Figures and Tables

**Table 1:** Correlation table for cubane-1,4-dicarboxylic acid, C_10_H_8_O_4_ (space group *P*2_1_/c, no. 14).

Free Molecule		Crystal	
(*C*_2h_)		Site ^1^ (*C*_i_)	Factor group (*C*_2h_, Z = 4)
Rep.^2^		Rep.	
External	Internal		
*A*_u_ + 2 *B*_u_ (trans)		3 *A*_u_	6 *A*_u_ + 6 *B*_u_
*A*_g_ + 2 *B*_g_ (lib)		3 *A*_g_	6 *A*_g_ + 6 *B*_g_
	19 *A*_g_	19 *A*_g_	38 *A*_g_ + 38 *B*_g_
	12 *A*_u_	12 *A*_u_	24 *A*_u_ + 24 *B*_u_
	11 *B*_g_	11 *A*_g_	22 *A*_g_ + 22 *B*_g_
	18 *B*_u_	18 *A*_u_	36 *A*_u_ + 36 *B*_u_

^1^ Symmetry of the site occupied by the molecule in the crystal. ^2^ Rep. = irreducible representation of the point group; trans = translation; lib = libration.

## Data Availability

The original data presented in this study are openly available in eData, the STFC Research Data Repository at https://doi.org/10.5286/edata/963 (accessed on 1 December 2025).

## Abbreviations

The following abbreviations are used in this manuscript:

INS	Inelastic neutron scattering
DFT	Density functional theory
